# Dietary Polyphenols and Gene Expression in Molecular Pathways Associated with Type 2 Diabetes Mellitus: A Review

**DOI:** 10.3390/ijms21010140

**Published:** 2019-12-24

**Authors:** Gideon Gatluak Kang, Nidhish Francis, Rodney Hill, Daniel Waters, Christopher Blanchard, Abishek Bommannan Santhakumar

**Affiliations:** 1 Australian Research Council (ARC) Industrial Transformation Training Centre (ITTC) for Functional Grains, Graham Centre for Agricultural Innovation, Wagga Wagga, NSW 2650, Australia; gkang@csu.edu.au (G.G.K.); nfrancis@csu.edu.au (N.F.); dwaters@csu.edu.au (D.W.); cblanchard@csu.edu.au (C.B.); 2 School of Biomedical Sciences, Charles Sturt University, NSW 2650, Australia; rhill@csu.edu.au; 3 School of Animal and Veterinary Sciences, Charles Sturt University, NSW 2650, Australia

**Keywords:** polyphenols, type 2 diabetes, pancreatic β-cell function, insulin resistance, gene expression

## Abstract

Type 2 diabetes mellitus (T2DM) is a complex metabolic disorder with various contributing factors including genetics, epigenetics, environment and lifestyle such as diet. The hallmarks of T2DM are insulin deficiency (also referred to as β-cell dysfunction) and insulin resistance. Robust evidence suggests that the major mechanism driving impaired β-cell function and insulin signalling is through the action of intracellular reactive oxygen species (ROS)-induced stress. Chronic high blood glucose (hyperglycaemia) and hyperlipidaemia appear to be the primary activators of these pathways. Reactive oxygen species can disrupt intracellular signalling pathways, thereby dysregulating the expression of genes associated with insulin secretion and signalling. Plant-based diets, containing phenolic compounds, have been shown to exhibit remedial benefits by ameliorating insulin secretion and insulin resistance. The literature also provides evidence that polyphenol-rich diets can modulate the expression of genes involved in insulin secretion, insulin signalling, and liver gluconeogenesis pathways. However, whether various polyphenols and phenolic compounds can target specific cellular signalling pathways involved in the pathogenesis of T2DM has not been elucidated. This review aims to evaluate the modulating effects of various polyphenols and phenolic compounds on genes involved in cellular signalling pathways (both in vitro and in vivo from human, animal and cell models) leading to the pathogenesis of T2DM.

## 1. Introduction

In 2017, an estimated 850 billion USD of global health expenditure attributed to treatment or other health interventions for diabetes. Present estimates show that 452 million people worldwide are affected by diabetes [1]. This number is expected to rise to 693 million by 2045, raising the cost to 958 billion USD [2]. Diabetes mellitus (DM) is a complex metabolic disorder characterised by insulin deficiency (in type 1 diabetes, an autoimmune disease), and insulin resistance or insulin deficiency attributable to other pathological pathways (in type 2 diabetes).

Type 2 diabetes mellitus (T2DM) is associated with several factors including hypertension, chronic hyperglycaemia and hyperlipidaemia, resulting from insulin resistance or insulin deficiency [3]. These factors have been implicated with overproduction of reactive oxygen species (ROS) in the mitochondrial matrixes that offset cellular redox balance and induce oxidative stress [4]. Excessive ROS-induced oxidative stress exerts significant damage to various cellular biomolecules including lipids, proteins and DNA [5]. The resulting dysregulated expression in various genes leads to impaired insulin secretion and impaired insulin signalling. This may induce advanced complications of T2DM such as hypertension and cardiovascular diseases, collectively known as metabolic syndrome [6,7].

**Figure 1:**
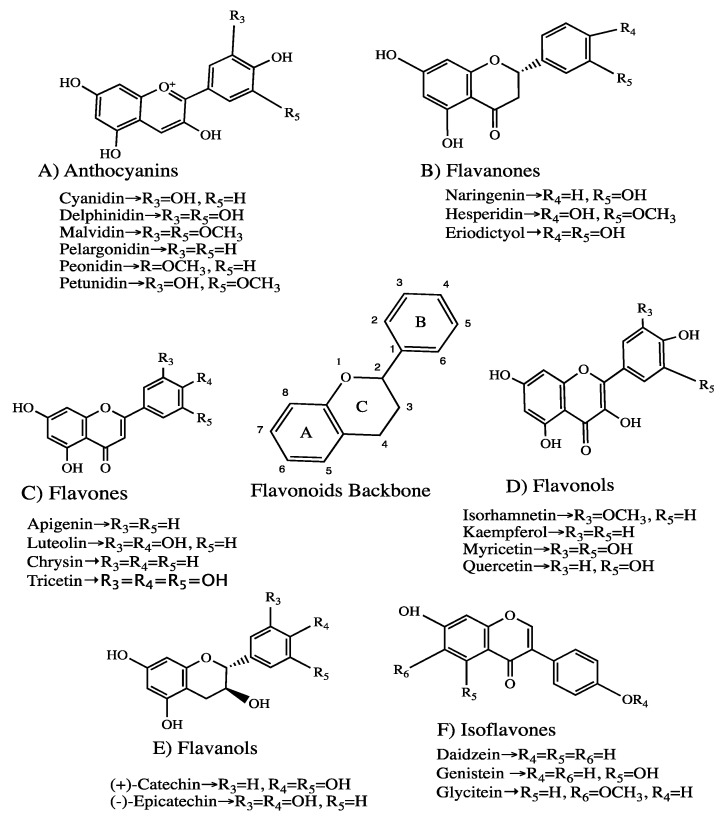
Common structures of flavonoid classes and their derivative compounds (Chemical structures are drawn using ChemDraw software). (**A**) Anthocyanins; (**B**) Flavanones; (**C**) Flavones; (**D**) Flavonols; (**E**) Flavanols; (**F**) Isoflavones.

Plant-based products, containing polyphenols, have demonstrated remedial benefits by reversing the metabolic processes of T2DM [8]. Dietary polyphenols and phenolic compounds (Figure 1) including resveratrol, γ-oryzanol, and epicatechins have been shown to regulate the expression of genes involved in insulin secretion (e.g., Sirtuin1 [Sirt1] and glucose transporter 2 [Glut2]) in β-cells [9] and insulin signalling mechanisms (e.g., glucose transporter 4 [Glut4] and peroxisome proliferator-activated receptor gamma [PPARγ]) in adipocytes [10]. Various polyphenols have also been shown to modulate the expression of insulin receptors substrate 1 (IRS-1), serine/threonine protein kinase 1 (Akt 1) and phosphoenolpyruvate carboxykinase (PEPCK) in human hepatoma cells (HEPG2) [11]. The positive effects of polyphenols on these pathways are correlated with improved β-cell function, insulin sensitivity, reduced inflammation and lipotoxicity and reduced hepatic glucose output, collectively accounting for normal glucose homeostatic function [12]. This review discusses the impact of polyphenols on regulating gene expressions in major metabolic pathways associated with the development of T2DM. Furthermore, the phenolic structures, the bioavailability and the potential mechanisms employed by polyphenols to mitigate the pathogenesis of T2DM are also discussed.

## 2. Pathogenesis of Type 2 Diabetes Mellitus

Type 2 diabetes mellitus is a multifaceted disorder with various contributing factors including genetics, epigenetics, environmental factors and lifestyle, particularly hypercaloric diets [13]. These risk factors affect the expression of genes involved in insulin secretion in β-cells and insulin sensitivity across peripheral tissues. Hyperglycaemia, a hallmark of T2DM, and hyperlipidaemia cause the overproduction of ROS and reactive nitrogen species (RNS), which then induce oxidative stress [14]. Reactive oxygen species/RNS-induced oxidative stress affects normal cellular metabolism of carbohydrates, proteins, fats and electrolytes, leading to genome and epigenome instability, cellular damage, inflammation and impaired organ function [15]. In particular, pancreatic β-cells are believed to have low endogenous antioxidant capacity, making them highly susceptible to oxidative stress [16]. Therefore, excess ROS production in β-cells leads to insufficient insulin secretion (β-cell dysfunction). In hyperlipidaemia, triglyceride and fatty acid-induced ceramide synthesis, coupled with excess nitric oxide (NO) production, initiates β-cell apoptosis and impair insulin secretion [17].

**Figure 2:**
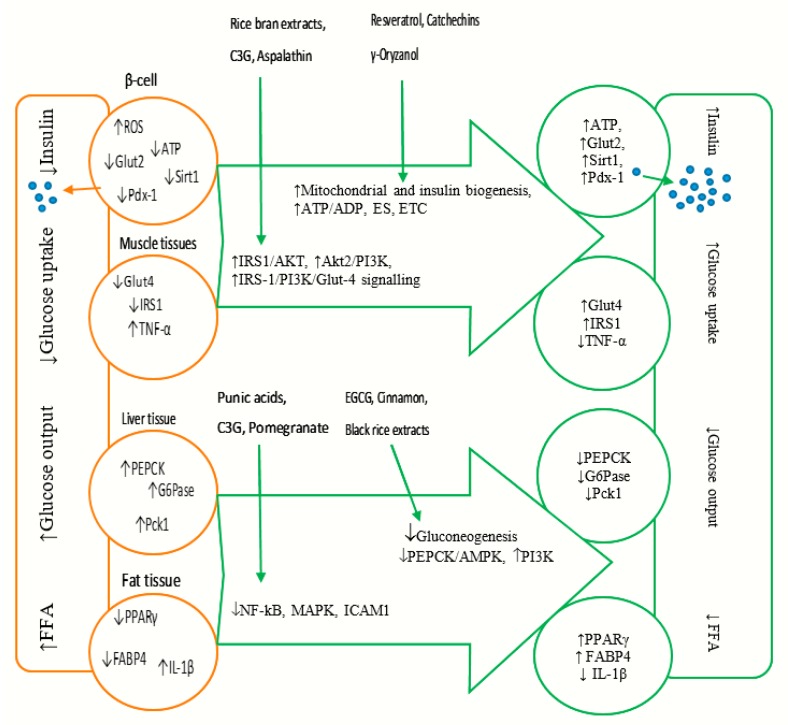
Schematic summary of how various plant-derived phenolic compounds target signalling pathways on various cell types and tissues in type 2 diabetes mellitus. ROS—reactive oxygen species; ATP—Adenosine triphosphate; Glut2—glucose transporter 2; Glut4—glucose transporter 4; Sirt1—Sirtuin 1; Pdx-1—pancreas and duodenal homeobox 1; IRS1—insulin receptor substrate 1; TNF-α—tumour necrosis factor alpha; ADP—Adenosine diphosphate; ES—endoplasmic reticulum stress; AKT—Protein kinase B; PI3K—Phosphatidylinositol 3-kinase; PEPCK—Phosphoenolpyruvate carboxykinase; G6Pase—Glucose-6-phosphatase; PcK1—Phosphoenolpyruvate carboxykinase 1; PPARγ—peroxisome proliferator-activated receptor; FABP4—fatty acid binding protein 4; IL-1β—Interleukin 1β; NF-kB—Nuclear factor kappa light chain enhancer of activated B cells; MAPK—mitogen activated protein kinase; ICAM1—intracellular adhesion molecules-1; C3G—Cyanidin 3-glucoside; EGCG—Epigallocatechin gallate; black arrows: ↑—increased gene expression and ↓—decreased gene expression); orange arrow—decreased insulin secretion under hyperglycaemic condition; short green arrow—increased insulin secretion after polyphenols treatment; long green arrows—different polyphenol extracts and phenolic compounds targeting various pathways.

In insulin-responsive cell types, ROS-induced oxidative stress is one of the mechanisms that disrupt insulin signalling and reduce insulin-stimulated glucose uptake in the targeted tissues such as skeletal muscle, liver and adipose. This leads to the development of insulin resistance [18]. Reduction of glucose uptake by insulin-responsive tissues leads to hyperglycaemia. In the liver, insulin resistance activates glucose production referred to as hepatic gluconeogenesis [19]. Consequently, this leads to excessive hyperglycaemia and elevated free fatty acid (FFA), furthering cellular oxidative stress-induced damages and T2DM complications [20]. However, various polyphenols have been shown to have antioxidant properties that may mitigate the adverse effects of hyperglycaemia in T2DM (Figure 2).

## 3. Polyphenol Classes and Their Structures

Polyphenols represent a diverse group of plant products widely found in vegetables, fruits, tea, coffee, cereals, chocolate, oils and various cocoa products [21,22]. The main classes of polyphenols comprise flavonoids (Figure 1) and non-flavonoids [23]. The major subclasses of flavonoids include anthocyanins, flavanones, flavones, flavonols and isoflavonoids [24]. Based on their phenolic rings and the structural elements binding the rings, dietary polyphenols are categorised into glycones (the sugar-phenolic group) and aglycones (the non-sugar group) [25]. Apart from flavanols, which are usually found as aglycones, most dietary polyphenols are glycones with the hydroxyl group conjugated by one or more sugar residues.

### 3.1. Flavonoids

Anthocyanins are glycosides characterised by aglycone connected to a sugar through a glycosidic bond. These are classified into cyanidin, delphinidin, petunidin, peonidin, malvidin and pelargonidin (Figure 1A). Recent investigations reported that red raspberries display a high content of cyanidin-3-*O*-sophoroside, cyanidin-3-*O*-(2″-*O*-glucosyl) rutinoside, cyanidin-3-*O*-glucoside, and cyanidin-3-*O*-rutinoside [26].

Flavanones are the most diverse flavonoid group characterised by the presence of a chiral centre at C-2 and the absence of a C2-C3 double bond (Figure 1B). The naturally occurring flavanones in plants include C-glycosyl, hydroxy, methoxy and methylenedioxy derivatives, with the C-ring attached to the B-ring at C-2 position [27]. Flavanone glycosides comprise hesperidin, narirutin and naringin. In addition to their aglycones (isosakuranetin, hesperetin, naringenin, and eriodictyol) these are found in citrus fruits such as oranges, tangerines and tangelos [28].

Flavones contain a double bond between C2 and C3, with B-ring attached at C2, as well as O- and C-glycosylation, O-methylation and hydroxylation. Except for the lack of oxygenation at C-3, flavone structures resemble flavonols and are mainly found as C-7-*O*-glycosides [29]. These include chrysin, acacetin, hispidulin and tricin (Figure 1C), which are usually found in celery, parsley, and some other herbs.

Flavonols are conjugated glucosides with sugar attachments at the 5, 7, 3′, 4′ and 5′ positions (Figure 1D). They are structurally identified by a 2-phenylchromen-4-one (2-phenyl-1-benzopyran-4-one) skeleton and represented by quercetin, kaempferol, myricetin and isorhamnetin. Berry, broccoli, onion together with tea and red wine are significantly rich in quercetin-4′-*O*-glucoside, quercetin-3, 4′-O-diglucoside and quercetin-3-*O*-rutinoside [30].

The most complex subclasses of flavonoids are flavan-3-ols, structurally ranging from simple monomers to the oligomeric and the most condensed tannins. The monomeric flavan-3-ols, contain two chiral centres at C2 and C3 (Figure 1E), produce four isomers representing each level of B ring hydroxylation among which (+)-catechin and (−)-epicatechin are naturally widespread [31].

Isoflavones are distinguished by the presence of the B-ring connected at C-3 instead of C-2 position (Figure 1F). The main compounds of isoflavones include daidzein, genistein and glycitein, which are exclusively found in leguminous plants such as soybeans. They are structurally identified as 7-*O*-(6″-*O*-malonyl)glucosides, 7-*O*-(6″-*O*-acetyl)glucosides, 7-*O*-glucosides, or simple aglycones [32].

### 3.2. Non-Flavonoids

These include phenolic acids, stilbenes and lignans; where phenolic acids are the most common group in human diets. Phenolic acids, derived from benzoic acid and hydroxycinnamic acid, are usually conjugated with one or more OH in the aromatic ring. Phenolic acids formed by C_6_ carbon skeleton are referred to as simple phenols (e.g., thymol and phenols cresol) while the non-simple phenols may be formed by C_6_-C_1_ carbon configuration such as gallic, syringic and vanillic acids. Other structural formations may involve C_6_-C_2_ (e.g., phenylacetic acids and acetophenones), C_6_-C_3_ (ferulic and caffeic acids) or aldehydes (e.g., vanillin) [27]. Stilbenes are phytoalexins compounds identified by C_6_-C_2_-C_6_ carbon structure. These are extremely low in human diets, thus, resveratrol (3,5,4′-trihdroxystilbene) is the main dietary compound identified (containing cis and trans isomers) conjugated with trans-resveatrol-3-*O*-glucoside (trans-piceid) derivatives. These are found in red wine, however, in very low concentrations [33]. It is argued that the major functions of phenolic compounds, such as antioxidant properties, metabolic activities and interactions with cellular receptors and enzymes, are governed by their chemical structures [34]. Additionally, the rate and extent to which these molecules are absorbed in the intestine and their bioavailability are also determined by their structural configurations [35].

### 3.3. Bioavailability of Polyphenols

Bioavailability has been referred to as the fraction of a substance, after which, when orally ingested, is absorbed and becomes available for physiological function or storage [36]. Dietary polyphenols are naturally occurring compounds found in plants and are usually consumed through foods. Before their bioactivities can be realised, polyphenols must first be absorbed, metabolised and then made available in human systems. The rate and the extent to which polyphenols are absorbed and further metabolised are influenced by several factors including their chemical structures, metabolic processing and the degree of conjugation [22]. During the course of absorption, aglycones (compounds not linked to glucose moiety) such as catechins, flavanols and flavones are absorbed in the small intestine and transported via the circulatory system into various tissues [29] where they elicit various biological effects. However, polyphenols linked to a glucose or rhamnose moiety forming ester, glycosides or polymers cannot be absorbed in their natural forms. They are usually transported to the colon and hydrolysed by gut microflora and digestive enzymes such as lactase phloridzin hydrolase (LPH) and rhamnosidases [37]. This results in the production and release of aglycones and various metabolites with diverse physiological functions [38].

Due to the nature of structural variations, the plasma concentrations of polyphenols vary greatly from one group or compound to another (Table 1). The protective properties of polyphenols depend on their bioavailability, thus evidence of their absorption may possibly relate to antioxidant activities in the plasma after consumption of polyphenol-rich foods [34].

It has been shown that the highest concentrations measurable in plasma following the consumption of dietary-rich polyphenols are in the nanomolar (nM) range, peaking at 2–4 h postprandially and are then rapidly excreted [48]. Others argue this concentration may be too low to elicit a measurable biological effect [49]. However, the efficacies of phenolic bioactivities may be influenced by temporal factors such as their half-life in the circulation and other factors such as the bioactivity of their metabolic degradation products. Although the exact half-lives of polyphenols have not been precisely determined, some compounds such as anthocyanins and flavanols were shown to last 2–3 h in the plasma [50]. Epigallocatechin gallate, however, is an exception as it is usually eliminated slowly due to high levels of complexing with plasma proteins. When considering isoflavones and the lignan group, longer half-lives of 6–8 and 9–24 h, respectively, were recorded (Table 1), perhaps contributing to their potent diabetic mitigating properties. As this evidence (Table 1) suggests, maintaining physiological concentrations of polyphenols in the plasma may require frequent consumption of polyphenol-rich diets, especially for those compounds that are rapidly absorbed and excreted.

### 3.4. The Proposed Mechanisms of Phenolic Action

The diabetic mitigating properties of polyphenols have been linked to their antioxidant and anti-inflammatory capabilities, thus, making them the central focus in the early herbal medicine practices for curing diseases [51]. However, the exact mechanisms driving these properties and how polyphenols can modulate cellular signalling pathways to reverse disease processes have not yet been properly elucidated [52]. Nevertheless, some potential mechanisms have been proposed.

#### 3.4.1. Interaction with Cell Membrane and Receptors

One of the important mechanisms by which polyphenols may elicit their biological effect is through initiation of cell signalling responses and interactions with both extracellular and intracellular receptors. Most polyphenols contain both hydrophilic and hydrophobic domains allowing their localisation and interactions with membrane components at different levels thereby generating cellular responses [53]. The polyphenol-membrane interaction then can induce membrane-associated functional changes such as modulation of signal transduction, ion metabolite flux, ligand-receptors interactions and membrane-associated enzymes activity [54]. For example, epigallocatechin gallate (EGCG) has been shown to regulate the activities of cell surface receptor tyrosine kinases (RTK), including insulin receptors (InsR) and insulin-like growth factor receptor (IGFR), by inhibiting tyrosine phosphatases [55,56], which in turn activates tyrosine phosphorylation and represses hepatic glucose output [57]. Furthermore, membrane localisation places polyphenols in close proximity with hydrosoluble and lipid-soluble radicals and as such, execute radical scavenging mechanisms [58].

Following membrane adsorption, it is claimed that polyphenols pass through membrane layers and internalise in the cytoplasm. A study on human colon adenocarcinoma cells (HT-29) demonstrated that after 15 min of incubation with a probed EGCG (0.5–20 µM), 75% of radioactively labelled EGCG was found in the cytosolic compartment with some activities also observed on the membrane fraction [59]. This indicates that polyphenols such as EGCG can directly bind to cell membrane components (lipids and proteins) and passively transfuse into the inner membrane where they interact with intracellular molecules and activate other pathways. However, other studies indicated that different phenolic compounds such as resveratrol can enter the cell through active transport, suggesting endocytosis via lipid raft [60]. This suggests that different phenolic compounds may have different mechanisms of cellular interactions based on their structural configurations.

#### 3.4.2. Metal Chelating Antioxidant Properties

The antioxidant characteristics of polyphenols have primarily been attributed to their ability to regulate the production of free radicals such as RNS and ROS [61]. Some polyphenols including flavonoids are known for their metal sequestration abilities, which prevent metal-catalysed free radical formation [58]. This is due to the high affinity for metal ions of catechol moieties of the B-ring and some structural elements, such as 6,7 hydroxyl and 4-carbonyl carbonyl groups, presented in various phenolic compounds [62]. Through this binding, flavonoids can thermodynamically neutralise highly oxidising species including superoxide, peroxyl and alkoxyl free radicals [63]. This reduces the adverse effect of oxidative stress and restores cellular redox status. In addition to their free radical scavenging properties, polyphenols have been shown to play an important role in modulating the expression of genes associated with the development of type 2 diabetes.

## 4. Effects of Polyphenols on Gene Modulations in T2DM

### 4.1. Polyphenols and Gene Modulations on β-Cell Dysfunction

When considering insulin secretion pathways, the normal cellular processes leading to insulin release are regulated under tight coupling between glucose metabolites (pyruvates, citrate, malate and glutamate) and nucleotides (ATP, NADH and NADPH). Mitochondria play a focal role in linking the proximal glycolytic and distal exocytosis events [64]. The proximal glycolytic process involves a potential gradient entry of glucose molecules into the cytoplasm through glucose transporter 2 (Glut2). Glucose is further phosphorylated to produce pyruvate, which is transported into mitochondria, leading to the production of ATP [65]. During this process, ROS are produced by NADPH oxidase as by-products following electron transfer from complex II to complex III [66]. Elevated ATP raises the ATP/ADP ratio and the closure of the cellular K_ATP_-channels, which in turn induces cell membrane depolarisation and the opening of Ca^2+^ channels [67]. Influx of Ca^2+^ increases cytosolic Ca^2+^ concentration, which then activates insulin exocytosis [68].

Under hyperglycaemic conditions, however, excess glucose-derived pyruvate transferred to tricarboxylic acid (TCA) cycle increases NADH/FADH_2_ influx into the mitochondrial electron transport chain and subsequently increases ROS production [69]. In hyperlipidaemia, elevated FFA levels lead to both FFA and acetyl coenzyme A (CoA) oxidation in the TCA cycle. This increases NADH/FADH_2_ donation into the electron transport chain resulting in ROS overproduction and oxidative stress. Increased levels of ROS can cause intracellular mitochondrial damage by inducing the opening of mitochondrial permeability transition and depolarisation. As a result, endogenous antioxidants leak out of mitochondria leading to mitochondrial depletion and apoptosis [70]. Mitochondrial damage reduces the ATP/ADP ratio hindering membrane depolarisation and the opening of Ca^2+^ channels. Consequently, this leads to delayed and insufficient insulin secretion. However, various polyphenolic compounds have been shown to neutralise oxidative stress by modulating the expression of genes along these pathways and thus improving insulin secretion and some of these extensively studied polyphenols are discussed further below.

Resveratrol belongs to the stilbene polyphenol group (found in grapes, berries and red wine) and possesses diverse diabetic mitigating properties. One of its major bioactivities has been implicated in improving pancreatic β-cell function and glucose homeostasis [71]. Treatment of rat insulinoma cells (INS-1E) with resveratrol (25 µM) upregulated the expression of some key genes for β-cell function such as Glut2, mitochondrial transcription factor (*Tfam*), pancreatic and duodenal homebox 1 (*Pdx1*), glucokinase (GK) and insulin 1 (*Ins1*) through the regulation of a master gene, *Sirt1* [9]. These effects potentiated a prominent glucose-stimulated insulin secretion (GSIS) response concomitantly with increased glucose oxidation and ATP generation (Figure 2). In another study using both human (HP62) and mouse (β-Min6) pancreatic β-cells, supplementation with resveratrol (0.1 μM) and curcumin (1 ppm) was shown to inhibit the expression of phosphodiesterase genes (*Pde3b*, *Pde8a* and *Pde10a*) by activating the cAMP pathway, which then resulted in an increased insulin secretion and improved β-cell function [72]. However, data from human clinical studies have presented conflicting results. While clear diabetic inhibiting effects have not been detected in a study by Boet et al. [73], the beneficial activities of resveratrol in maintaining fasting blood glucose and reducing oxidative stress in diabetic patients has been reported by others [74]. Variation in these results may be attributed to the fact that these studies measured different parameters such as cholesterol level and other metabolic variables, suggesting resveratrol may have some specific β-cell activation properties.

Some of the flavanol compounds with potent diabetic reducing effects are catechins, which are mainly found in cocoa (Table 2). Previous studies have reported that catechins from cocoa-rich diets can attenuate β-cell mass loss and ameliorate β-cell function, by preventing oxidative stress and apoptosis in diabetic rats [75]. In a recent study using INS-1 832/13-derived β-cells, treatment with monomeric cocoa catechins (0.25 µg/mL) significantly increased insulin secretion by enhancing the expression of genes responsible for mitochondrial complex biogenesis such as Nuclear factor erythroid 2-related factor 2 (*Nrf2*) and *Nrf1*, and GA binding protein transcription factor alpha subunit (*GABPA*) [76]. In the same study, however, it was demonstrated that incubation of β-cells, INS-1 832/13, with catechins did not increase mitochondrial content or viability. This suggests that catechins-induced β-cell insulin secretion may not be due to mitochondrial population or increase in mitochondrial viability. As such, further studies are warranted to elucidate this mechanism.

Tyrosol is a well-known phenolic compound found in olive oil and white wine. Previous studies have reported a potent antioxidant activity by scavenging peroxynitrite and superoxide ions relieving cellular stress [82]. In tunicamycin-induced endoplasmic reticulum (ER) stress mouse insulinoma cells (NIT-1), treatment with tyrosol (25 and 50 µg/mL for 48 h) was shown to downregulate the expression of stress-related genes [binding immunoglobulin protein (*BIP*), inositol-requiring kinase 2α (*eIF2α*), C/EBP-homologous protein (*CHOP*) and protein kinase-like endoplasmic reticulum kinase (PERK)] by inhibiting the phosphorylation of c-Jun N-kinase (JNK) pathway [77]. Thus, the ER stress inhibition properties of tyrosol enhanced insulin production by improving β-cell mass and survival.

Extracts from jojoba seed, containing a wide range of phenolic compounds, demonstrated an active free radical scavenging ability leading to improved β-cell protection [83]. A study using rat cells (RINm5f) demonstrated that jojoba seed crude extracts significantly reduced ROS levels by 69% when compared to simmondsin, the major phenolic compound found in jojoba [78]. This pattern was also reflected in caspase activation, antioxidant activity and pro-oxidant signalling pathways, where the crude extracts significantly increased the expression of *Nrf2* while simmondsin displayed no effect. When considering p22phox, however, it was the simmondsin extracts that inhibited the activity of this gene. *Nrf2* plays a key role in antioxidant defence, while p22phox activates NADPH oxidase, generating excessive ROS [84,85]. The regulation of both genes by jojoba seed extracts preserved β-cell function demonstrated by increased insulin secretion [78].

γ-Oryzanol (Orz) is another example for a structurally unique bioactive phenolic compound (featuring a mixture of ferulic acid esters and phytosterols or triterpene alcohols) exclusively found in brown rice [86]. In a similar manner to tyrosol, the diabetic healing effects of Orz have also been linked to β-cell protection against ER stress-induced apoptosis. Supplementation with Orz (0.2 or 2.0 µg/mL) for 24 h reduced the expression of several ER stress signalling genes (ER resident DNAJ 4 (*ERdj4* or *Dnajb9*), spliced form of X box binding protein 1 (*Xbp1s*) and *CHOP*) and apoptosis responsive genes (caspase-3 (*Casp3*) and caspase-activated DNase [CAD]) in tunamycin-induced ER stress MIN6 cells [79]. This study also demonstrated that the same genes were downregulated in pancreatic islets of HFD-fed mice (C57BL/6J) treated with Orz (320 µg/g body weight [BW]) for 13 weeks, which positively correlated with improved β-cell glucose-stimulated insulin secretion (GSIS). Surprisingly, the same study claimed that Orz did not influence some of the key genes [*Pdx1*, *Mafa*, *Neorog-3* (*Ngn3*), *Ins1* and cyclin-dependent kinase inhibitor 1A (*Cdkn1a*)] that regulate β-cell survival and proliferation, suggesting a specific anti-stress related mechanism for Orz. The different effects observed by tyrosol and Orz on the same in vitro cell culture models suggest that each bioactive compound may act through various cellular signalling pathways to regulate β-cell function.

EGCG is the most abundant polyphenol in green tea and one of the most common flavanol compounds with diverse diabetic relieving functions including improved insulin secretion via enhanced β-cell viability and glucose uptake via effective insulin signalling [80]. In rat pancreatic cells (RIN-m5F), supplementation with EGCG (1–10 µM) for 2 h increased the expression of *Pdx-1*, Forkhead box O1 (*FOXO1*) and protein kinase B (Akt) phosphorylation, resulting in augmented β-cell viability and insulin secretion [80]. This suggests AKt/Pdx-1 pathway mechanism to maintain β-cells function. Furthermore, this study demonstrated that treatment with EGCG maintained the expression of *Pdx-1* (controls mitochondrial biogenesis) for up to 72 h in high glucose (33 mM) concentrations. Since EGCG is an aglycone molecule with maximum detectable plasma concentration only at 1 h (Table 1), gene modulating activities lasting for up to 72 h may indicate that EGCG possibly uses a positive-feedback mechanism to maintain protective effects long after its excretion.

*Centratherum anthelminticum* seeds are widely known for their hypoglycemic properties in treating diabetes. In an in vitro study, treatment of β-TC6 cells with (6.25–50 µg/mL) crude methanolic fraction of *C. anthelminticum* seeds (CAMFs) dose-dependently increased insulin secretion by enhancing the expression of Glut2, thereby improving β-cells function [81]. Looking at the in vivo effect, the same study reported that treatment of Sprague–Dawley rats with CAMFs (50 mg/kg body weight) reduced blood glucose levels in both type 1 and type 2 diabetic subjects. When considering insulin secretion, however, it was shown that CAMFs increased insulin secretion only in type 2 diabetic rats. These findings suggest that CAMFs may offer diabetic mitigating effects only by upregulating β-cells function genes rather than β-cell preservation.

### 4.2. Polyphenols and Gene Modulations on Insulin Signalling Pathways

The processes that govern insulin signalling (in insulin-responsive tissues) involves a series of activation cascades initiated by insulin binding to its receptor resulting in tyrosine phosphorylation of insulin receptor substrates (IRSs). This, in turn, activates phosphatidylinositol 3-kinase (PI3K) followed by subsequent phosphorylation events leading to the activation of Akt and Ras-mitogen-activated protein kinase (MAPK) in insulin responsive tissue types [87]. Activation of Akt/MAPK stimulates transcription factors triggering the translocation of insulin-mediated Glut4 to the plasma membrane, of muscle and adipose tissues, thereby allowing increased glucose transport into the cell [88]. However, under hyperglycaemic and elevated FFA conditions, it is believed that overproduction of ROS activates nuclear factor kappa light chain enhancer of activated B cells (NF-κB) and stress transduction pathways, such as JNK [89]. This then initiates serine phosphorylation of IRS-1 (pIRS-1(S307), thereby inhibiting Akt/PI3K activation and Glut4 translocation [90]. Consequently, this reduces insulin sensitivity and disrupts cellular glucose uptake, leading to insulin resistance.

In addition to insulin secretion activities, the diabetic reducing properties of catechins have also been related to improving insulin signalling mechanisms. In streptozocin-induced diabetic mice, three weeks of treatment with catechin (50 mg/kg/day) significantly enhanced PI3K and the endothelial nitric oxide synthase signalling system [91]. Consequently, this increased insulin sensitivity, improved glucose uptake, lowered serum glucose levels and prevented vascular endothelial dysfunction [92].

Numerous studies have reported that sweet potato extracts (SPE) have a higher polyphenolic content associated with important antioxidant functions [93]. Quantitative in vitro investigations demonstrated that treatment of palmitate-induced insulin-resistant mouse myoblast cells (C2C12) with extracts 500 µg/mL and 100 µg/mL of orange sweet potato tubers (OSPT) and leaves (OSPL), respectively, improved glutathione (GSH) status, increased antioxidant capacity and enhanced antioxidant enzyme (Gpx and CAT) activities [94]. These effects were shown to directly upregulate the expression of some key genes (*Glut4, Nrf1* and myocyte enhance factor 2A [*Mef2a*]) along the insulin signalling pathway. *Glut4* is the major insulin-sensitive transporter in both skeletal muscle and adipose tissues [95], and its downregulation in glucolipotoxicity is associated with severe insulin resistance [96]. The two transcription factors, *Mef2a* and *Nrf1*, regulate the expression of *Glut4* and, ultimately, glucose uptake metabolism [97]. In the same study, OSPT and OSPL also modulated the expression of carnitine palmitoyl transferase 1 (*CPT1*) and acetyl CoA carboxylase 2 (*ACC2*) genes, which are involved in the regulation of mitochondrial fatty acid oxidation and subsequently improve insulin sensitivity. Such genetic modulatory effects of sweet potato extracts indicate a remedial potential to improve insulin sensitivity in the targeted tissues.

The diabetic healing effects of plant-derived hormones (strigolactones) and stilbenoid polyphenol (pinosylvin) may be related to their ability to stimulate the *SIRT1* gene under diabetic conditions. *SIRT1* is a regulatory gene whose activation improves insulin sensitivity [98], mitochondrial biogenesis, energy metabolism and decreases obesity-induced inflammation [99]. Treatment of Rat L6 myoblasts with strigolactones and stilbenoid (60–100 µM) for 6 h enhanced glucose uptake (Table 3). Strigolactones stimulated SIRT1, insulin receptor substrate 1 (IRS-1), phosphatidylinositol-3-kinase (PI3K), *NRF1, Glut4* and *FOXO1* translocation, thereby increasing insulin signalling sensitivity and mitochondrial biogenesis [100]. The effect of pinosylvin, in this study, was minimal at the transcription level but it stimulated the phosphorylation of AMPK, suggesting energy metabolic pathway activities.

Another medicinal plant, *Hibiscus sabdariffa* L. calyx, has been known for its glucose-lowering ability. A study using human hepatocytes (HK-2) demonstrated that treatment with *Hibiscus sabdariffa* polyphenol extracts (up to 1 mg/mL) reduced pIRS-1 (S307) phosphorylation and inhibited dipeptidyl-peptidase-4 (DPP-4) by upregulating pPI3K [101]. DPP-4 is one of the gut enzymes responsible for breaking starch down to glucose during digestion, raising glucose levels [107]. Inhibition of this enzyme suppresses postprandial hyperglycaemia, and upregulation of pPI3K improves insulin signalling mechanisms, thus alleviating insulin resistance.

Anthocyanins (ACNs) are a group of flavonoids and the most widely consumed polyphenols (with a daily intake of 180–250 mg/day) found in fruits and berries [21]. Numerous investigations have reported diabetic reducing activities of ACN derivatives (cyanidin-3-*O*-β-glucoside (C3G) and protocatechuic acid (PCA)) by increasing insulin sensitivity and glucose uptake [108]. In both human and murine (3T3-L1) adipocytes, treatment with C3G and PCA (10–100 µmol/L) was shown to exhibit insulin-like properties by enabling Glut4 membrane translocation as well as upregulating PPARγ and the adiponectin gene [10]. PPARγ is a nuclear receptor that controls protein transcription in glucose and fatty acid uptake, whereas adiponectin, a hormone produced in adipocytes, has been referred to as an insulin sensitiser with both proteins involved in glucose and lipid metabolism [109]. Their expression, in this study, correlated with improved insulin resistance and lower blood glucose. However, inhibition of the PPARγ encoding gene counteracted the upregulation of *Glut4* and adiponectin by anthocyanins, suggesting a direct PPARγ regulatory mechanisms of these genes by C3G and PCA. Although C3G demonstrated better modulatory activities, both compounds displayed similar patterns of genetic regulation in both human and murine adipocytes, more effectively at 18 h incubation period.

Rice brans (RB), derived from the rice milling process, contain phenolic compounds including ferulic acid, sinapic acid and protocatechuic acid [110]. Pigmented rice brans are considerably rich in anthocyanins and proanthocyanidins such as cyanidin 3-glucoside (C3G) and peonidin 3-glucoside [111]. With diverse antioxidant and glucose homeostatic capabilities, diabetic reducing effects of rice bran are well documented [112,113]. Fermented RB (FRB) extracts have been associated with antioxidant and hypoglycaemic effects in T2DM [114]. Treatment of adipocytes (3T3-L1) with FRB extracts (10 μg/mL and 50 μg/mL) for 12 h significantly increased the expression of PPARγ and adiponectin by neutralising free radicals formed by high glucose (25 mM)-induced oxidative stress [103]. The same study also demonstrated that FRB extracts inhibited the expression of tumour necrosis factor-alpha (TNF-α), resulting in an increased insulin sensitivity, reduced insulin resistance and hyperglycaemia. The expression of *Glut4* in this study, however, was not changed by the treatment of FRB extracts, suggesting that glucose-lowering effects of FRB extracts may not be via direct activation of Glut4 translocation.

In contrast, pigmented rice bran extracts have demonstrated insulin-like activities both in vitro and in vivo [115]. Treatment of 3T3-L1 with (50 µg/mL) rice bran extracts from red and purple rice (*Oryza sativa* L.) for 8 and 12 h increased the expression of *Glut4*, *Glut1*, INSR, ISR1, Akt2 and PI3K, the key components in the insulin-signalling pathway [103]. This, consequentially, increased both insulin sensitivity and glucose uptake (Figure 2). Boue and colleagues demonstrated that pigmented rice bran extracts also inhibited the activities of key digestive enzymes (PPD-4, α-amylase and α-glucosidase), and this has been associated with suppressed postprandial hyperglycaemia [116]. Data from Boue and co-workers indicated that following an 8 h incubation, both red and purple rice bran extracts increased Akt2 expression 4.59-fold and 2.29-fold, respectively. After 12 h, however, this expression slightly reduced to 4.1-fold and 1.1-fold, respectively. Apart from the *Akt2* gene, the modulating effects of red bran extract were optimally exhibited after a 12 h incubation. With the exception of Akt2 and the glucose transporters, however, purple rice bran extract was observed to be more effective at 8 h incubation. When considering the bioavailability of anthocyanins (Table 1), it has been shown that these compounds may only stay in circulation for up to 1.11 h [45]. Based on this, the extended time response (12 h) reported by Boue and colleagues [103] suggest that other bioactive molecules may be responsible for the modulatory effects observed.

Aspalathin (ASP) derived from *Aspalathus linearis* is a dietary flavonoid with potent antioxidant capability and diverse diabetic inhibiting mechanisms. ASP exhibits a glucose-lowering effect comparable to metformin [117]. In rat diabetic cardiomyocytes (H9c2) exposed to 33 mM glucose, treatment with 1 µM ASP increased the expression of Glut4, ACC and uncoupling protein 2 (UCP2) by decreasing the expression of adenine monophosphate activated protein kinase threonine 172 (pAMPK (Thr172)) and CPT1 [70]. These effects concomitantly increased the level of antioxidant enzymes and anti-apoptotic genes (Bcl2/Bax ratio), which, as a result, improved cell viability, insulin sensitivity and glucose uptake.

*Molineria latifolia* is a perennial herbal plant with vital diabetic ameliorating properties. Findings from previous studies reported diabetic healing efficacy of *Molineria latifolia* crude extract by upregulating key genes (*IRS-1*, *Glut4* and *IGF-1*) in insulin-signalling pathways [118]. An investigation using high-fat diet (HFD)-induced diabetic animal models demonstrated that treatment of male rats with polyphenol-rich ethyl acetate fraction (100–200 mg/kg BW) from *Molineria latifolia* significantly increased the expression of insulin-signalling effectors such as IRS1, IRS2, Akt2 Glut4 and hexokinase 2 (HK2) through phosphorylation of IRS1/Akt pathway in skeletal muscle tissues [105]. This was associated with increased insulin sensitivity and improved glucose uptake, thereby reducing insulin resistance [119].

Mulberry leaf (*Folium Mori*) phenolic-rich extract is widely known for its diabetic mitigating properties and has been extensively used in Chinese medicines to treat diabetic symptoms such as high blood glucose, hyperlipidaemia and diabetic-induced nephropathy [120]. In treating STZ-induced diabetic rats with (2 g/kg BW) the extract was shown to increase the expression of insulin signalling genes (*IRS-1*, *PI3K*, and *Glut4*) in skeletal tissue, which, as a result, reduced insulin resistance effects, improved glucose tolerance and significantly reduced plasma glucose levels [106]. These findings suggest that *Folium mori* extract can upregulate insulin sensing genes and ameliorate insulin resistance by activating the IRS-1/PI3K/Glut4 pathway.

### 4.3. Polyphenols and Gene Modulations on Gluconeogenesis Pathways

In hepatocytes, elevated FFA levels lead to ectopic fat deposition (storage of triglycerides in tissue other than adipose tissues), which consequently inhibits IRS2-associated AKT/PI3K cascade activation and Glut2 expression, reducing insulin-stimulated glucose uptake (insulin resistance) [121]. Ectopic fat-induced inhibition of AKT/PI3K decreases the phosphorylation of *FOXO1*, which, as a result, activates the transcription of glucose-6-phosphatase (G6Pase) and phosphoenolpyruvate carboxykinase (PEPCK), the rate-limiting enzymes for gluconeogenesis [122]. The resulting increased hepatic glucose production leads to hyperglycaemia and the development of T2DM [123,124].

Cinnamon extracts (CE) have been reported to improve insulin sensitivity and glucose homeostasis by regulating hepatic enzymes activities, attributed to its phytochemical composition such as cinnamic acid, cinnamaldehyde and proanthocyanidins [125]. Supplementation of rat hepatoma cells (H4IIE) with (1–25 μg/mL) CE was demonstrated to inhibit hepatic glucose production by downregulating the expression of PEPCK and G6pase (Figure 2), concomitantly decreasing blood glucose levels [126]. Such insulin-like and glucose-lowering effects of CE may help to ameliorate T2DM conditions.

*Fructus corni* (*Cornus officinalis*) is another polyphenol-rich (loganin and ursolic) plant known for its stimulatory role of liver and glucose uptake diabetic lowering activities [127]. Treatment with 50 mg/mL of *Fructus corni* extracts demonstrated insulin-mimetic effects by inhibiting the expression of PEPCK [128] in the liver tissue, whereas, loganin and ursolic compounds failed to exhibit any effect on PEPCK expression. This suggests that these two major components may not be the only putative actives in the extract and that some other compounds may be responsible for the potent synergistic bioactive effects in the gluconeogenesis pathway.

In addition to its potent insulin secretion abilities, EGCG also inhibits glucose production in hepatocytes. Incubation of H4IIE cells with EGCG (5–25 µM) was shown to suppress *PEPCK* and *G6Pase* genes via PI3K activation in a dose-dependent manner [57], resulting in reduced hepatic glucose output (Figure 2). It was also shown, in the same study, that treatment of H4IIE with EGCG (up to 50 µM for 30–240 min) promoted tyrosine phosphorylation of insulin signalling proteins such as IR-β, IRS-1 and IGF-1R through Akt/PI3K activation, owing to its insulin-mimetic properties. On the contrary, later investigations argued that the suppression of glucose production by EGCG does not involve the activation of insulin signalling pathway, as inhibition of PI3K demonstrated no effects on the activities of EGCG [129]. Nevertheless, both studies concurred that EGCG suppression of hepatic gluconeogenesis was dependent on initial production of ROS, a known activator of Ca2+/calmodulin-dependent protein kinase kinase (CaMKK) [130] and tyrosine-phosphorylated proteins [131]. The exact mechanisms of how EGCG activates CaMKK via ROS production are, however, still unclear. As such, further investigations are warranted to identify molecular targets for the management of T2DM.

Extracts from germinated-brown rice arguably contain a higher amount of bioactive compounds than brown rice and have thus been known for their diverse diabetic lowering activities including blood glucose-lowering effects, improved total plasma cholesterol and enhanced hepatic function [132]. The diabetic mitigating properties of germinated-brown rice products have been related to the presence of bioactive compounds such as gamma-amino butyric acid, acylated steryl glycoside, oryzanol, and other phenolics [133]. A nutrigenomic investigation reported that exposure of hepatic cells (HEPG2) and diabetic rats (Sprague–Dawley) to 50 ppm and 50–100 ppm of germinated-brown rice extracts, respectively, downregulated hepatic gluconeogenic genes such as Fructose-1,6-bisphosphatase (*Fbp1*) and Phosphoenolpyruvate carboxykinase 1 (*Pck1*), more potently than metformin [134]. This, as a result, inhibited hepatic glucose output and reduced blood glucose levels, suggesting that germinated-brown rice extract may provide anti-hyperglycaemic properties by inhibiting hepatic glucose production and, therefore, help to manage T2DM.

Hesperidin and naringin are citrus flavonoids with implicated antioxidant capacity, lipid and glucose-lowering effects [135]. In hyperglycaemic-induced diabetic mice, treatment with hesperidin and naringin (0.2 g/kg BW) for 5 weeks downregulated G6Pase and PEPCK in liver tissue, thereby reducing blood glucose levels [136].

Coupled with its potent β-cell genes upregulation and insulin secretion ameliorating properties, resveratrol has also been shown to modulate the expression of genes controlling hepatic gluconeogenesis processes in T2DM (Table 4). Animal studies revealed that treatment of diabetic mice (C57BL/KsJ-*db*/*db*) with resveratrol (0.02%, *w*/*w*) for 6 weeks significantly decrease the expression of hepatic gluconeogenic genes *SREBP-1c*, *PECK* and *G6P* by activating AMPK and its downstream targets in liver tissues [137]. The resulting inhibition of hepatic glucose output was correlated with reduced blood glucose and plasma FFA levels and improved hepatic function. This suggests resveratrol as a potential agent to help manage T2DM through the activation of AMPK pathway.

Fermented food paste (FFP) is a source of polyphenols, including caffeolyquinic acid and sakuranetin, with potential diabetic relieving properties associated with its hyperglycaemic regulatory role and hepatic protective mechanisms [138]. Supplementation of streptozotocin (STZ)-induced diabetic mice with FFP (0.1 and 1.0 g/kg BW) for 6 weeks upregulated the expression of *GK*, phosphofructokinase (*PFK*), and 6-phosphogluconate dehydrogenase (*6PGD*) genes in the liver tissue, responsible for hepatic glycolysis (breaking down of glucose to ATP) [138]. Such modulatory effects were correlated with hepatic insulin sensitivity and glucose uptake, inhibiting hepatic gluconeogenesis. Besides glucose output inhibit, FFP was also shown (by the same study) to increase the expression of glucose transporter genes (*Glut1*, *Glut4*, and *Glut8*) and adiponectin gene, but inhibits pro-inflammatory cytokines (IL-1β and TNF-α). This may suggest diverse diabetic lowering activities of FFP, indicating a potential for glucohomeostasis management in T2DM.

### 4.4. Effects of Polyphenols on Lipid Peroxidation Pathways

Excessive FFAs has been associated with increased ROS, which through various molecular receptors, attach to cell membranes and initiate progressive lipid oxidation to form lipid peroxide. This mechanism renders the double bonds of polyunsaturated fatty acids unstable leading to cellular injury, thereby disrupting cellular signalling mechanisms controlling glucohomeostasis [139,140]. Various polyphenols and phenolic compounds have been shown to possess adipogenic gene modulatory effects attributed to their antihyperlipidaemic properties.

Consumption of pomegranate (*Punica granatum* Linn), rich in phenolic compounds such as ellagic acid, flavonoids, anthocyanins and punicalagin [141], has been linked to an increased level of antioxidant enzyme activities, reduced lipid peroxidation and improved glucose homeostasis [142,143]. In vitro study using liver cells (HepG2) and in vivo study using male Zucker diabetic rats demonstrated that pomegranate flower extract upregulated key adipogenic genes *PPARα*, carnitine palmitoyltransferase-1 (*CPT-1*) and acyl-CoA oxidase (*ACO*), while *PPARγ* remained unchanged in both HepG2 and liver tissue from the diabetic rats [144]. This substantially reduced triglycerides level and lipid accumulation in the liver (ectopic lipid storage), thereby improving hepatic glucose metabolisms.

Punicic acid (PA) from pomegranate was shown to enhance the expression of PRAR α and γ, and fatty acid-binding protein 4 (FABP4) in muscle cells of *db/db* mice, increasing insulin sensitivity [145]. FABP4 is involved in glucose and lipid metabolism, and its upregulation has been associated with the development of insulin resistance and T2DM [146]. As such, its upregulation reported here may need further investigations. Supplementation of 3T3-L1 with PA (30 µM) increased the expression of *PPARγ*, *Glut4* (Figure 2) and reduced ROS [147]. However, in recent studies using an adipocyte cell line (3T3-L1), PA and its main polyphenols (ellagic acid, punicalagin and the gastrointestinal metabolite urolithin A) downregulated *PPAR-γ*, *Glut4* and *FABP4* [148,149]. Although variations in study design and cell models may contribute to the conflicting results, pomegranate extracts may upregulate the expression of adipogenic genes to enhance free fatty acid uptake into the targeted tissues (muscles, liver and adipose tissues) while inhibiting their expression in adipocyte saturated tissues (intestines). Besides genetic modulation, pomegranate extracts have also been shown to inhibit the activities of key enzymes linked to T2DM (DPP-4, α-glucosidase and lipase) and reduced lipid accumulation in 3T3-L1 adipocyte-like cells [148].

### 4.5. Effect of Polyphenols on Inflammatory Pathways

Under chronic glucolipotoxicity, overproduction of ROS leads to protein oxidation which consequently generates pro-inflammatory signals (peroxiredoxin 2). This triggers an inflammatory response recruiting M1 macrophages activated via toll-like receptor-4 (TLR-4) to produce pro-inflammatory markers such as TNF-α, interleukin 1β (IL-1β), IL-6 and MCP1 [150]. IL-6 and TNF activate the Janus kinase/signal transducer and activator of transcription (JAK/SAT) and mitogen-activated protein kinase (MAPK), respectively, inducing cellular damage and insulin resistance. Lines of evidence have reported that polyphenols can downregulate key inflammatory markers such as protein kinase-c, cyclooxygenase-2 (COX-2) and inhibit major mediating pathways such as NF-κB, inducible nitric oxide synthase (iNOS) and MAPK [151]. Phenolic compounds have also been shown to modulate inflammatory signalling processes by altering enzymatic activities such as tyrosine and serine-threonine protein kinase.

Punicic acid (PA) from pomegranate has also been shown to possess anti-inflammatory activities by suppressing the expression of pro-inflammatory molecules in addition to its enhanced lipid metabolic activities. In obese diabetic mice, treatment with 5 and 10 μM PA suppressed NF-κB and TNF-α in both adipose and liver tissue, in a dose-dependent manner [145]. Coupled with its insulin signalling ameliorating effects, as mentioned above, ASP has also been proven to downregulate inflammatory genes suppressor of cytokine signalling 3 (*Socs3*), tumour necrosis factor receptor superfamily (*Tnfsf*), CD44, JAK/STAT and MAPK) and pro-apoptotic genes (*Mapk3*, optic atrophy 1 (*Opa1*) and *Chuck*) by neutralising intracellular ROS and reducing DNA nick formation [104].

Besides their effective insulin signalling proficiency, the diabetic reducing behaviours of anthocyanins have also been attributed to their anti-inflammatory and anti-oxidative functions. Treatment of diabetic cells (HK-2) with anthocyanins derivatives C3G and cyanidin chloride (Cy) at 50 µM markedly increased the expression of *PPRARα* and *LXRα* genes [152]. This caused the downregulation of hyperglycaemic-induced pro-inflammatory cytokines namely intracellular adhesion molecule-1 (ICAM1), transforming growth factor-β1 (TGFβ1) and monocyte chemoattractant protein-1 (MCP-1) by inhibiting the NF-κB pathway (Figure 2). Another study (on diabetic primary HK-2 cells) supporting these findings demonstrated that supplementation with anthocyanins (10 µM) suppressed the expression of apoptosis-related genes (thioredoxin interacting protein (TXNIP), Bcl-2, caspase-3 and ROS) by inhibiting p38MAPK and ERK1/2 phosphorylation [153]. The resulting decrease in cellular glucose level has been associated with improved diabetic nephropathy, a primary complication of T2DM [154].

ROS-induced inflammation is also linked with epigenetic modifications, leading to the generation of cytokines. The flavonoid fisetin, commonly found in fruits and vegetables, has been known to offer diabetic healing effects by regulating histone deacetylases (HDACs) activities. In hyperglycaemic human monocytes (THP-1), treatment with fisetin (3–10 µM) was reported to activate HDACs and downregulated histone acetylates (HAT) activities, thereby inhibiting NF-κB pathways and suppressing cytokine release [155]. In another study using THP-1 cells, (−)-Epicatechin (EC), a major compound in flavanols, was shown to mitigate hyperglycaemic-induced histone acetylation by initiating chromatin remodelling which prevents p65-NF-κB binding to the TNF-α promoter thereby inhibiting its expression [156]. This resulted in decreased cytokine release and improved glucose uptake. Adding to its spicy nature, curcumin (found in turmeric) has been suggested to exert potent anti-inflammatory effects [157], validating the reasoning for seasoning. In THP-1 cells (exposed to 25 mM glucose), supplementation with curcumin (1.5–12.5 µM) significantly downregulated HAT activity, P300 level and *CBP*/*p300* gene expression by suppressing NF-κB binding to TNF-α [156].

## 5. Conclusions

T2DM is a multifaceted disease with various contributing factors including over-nutrition and genetic dysregulation, leading to insulin deficiency (also referred to as β-cell dysfunction) and insulin resistance. Over-nutrition contributes to hyperglycaemia and hyperlipidaemia generating oxidative stress, which, as a result, induces cellular metabolic dysregulations. This affects gene expression in major pathways controlling glucohomeostasis. Polyphenolic compounds have antioxidant properties and can modulate the expression of genes along these pathways to mitigate the diabetic effects (Figure 2). The exact mechanisms of action of polyphenols are not well understood, but polyphenol structural elements play a significant role in relation to their interactions with other proteins, absorption, transportation and bioavailability. Further studies are warranted to identify polyphenols with specific functions and thus may offer a therapeutic remedy for the management of T2DM.

## Figures and Tables

**Table 1:** Bioavailability of polyphenols in human plasma. EGCG—epigallocatechins Gallate; EC—epicatechins; Conc.—concentration.

Polyphenols	Plasma Conc. (C_max_)	Half-Life (T_1/2_)	Quantities	Food Source	Ref.
Quercetin	0.3–0.75 µmol/L	0.6 h	80–100 mg	Onion	[39]
EGCG and EC	0.1–0.7 μmol/L	1 h	90–150 mg	Green tea	[40]
Epichatechin	0.25–0.7 μmol/L	2 h	70–165 mg	Cocoa	[41]
Catechin	0.09 μmol/L	1 h	35 mg	Red wine	[42]
Hesperetin	1.3–2.2 μmol/L	5–7 h	130–220 mg	Orange	[43]
Naringenin	6 μmol/L	5–7 h	200 mg	Grapefruit	[44]
Anthocyanins	97.4 nmol/L	1.11 h	110–200 mg	Elderberry extracts	[45]
Lignan	30 nmol/L	9–24 h	25 mg	Linseed	[46]
Isoflavones	1.4–4 μmol/L	6–8 h	50 mg	Soy	[47]

**Table 2:** Polyphenols and gene modulations on β-cell function pathways.

Polyphenols/Conc.	Genes Affected	Function	Pathways	Cells/Tissue Type	Ref.
**In vitro models**					
Resveratrol (25 µm)	↑ *SIRT1*, ↑ *Glut2*, ↑ *GK*, ↑ *Pdx-1*, ↑ *Hnf-1α*, ↑ *Tfam*	↑ Insulin biogenesis	Mitochondrial	Cells-INS-1E	[9]
Resveratrol (0.1 µM) and curcumin (1 PMOL/l)	↑ cAMP ↓ PDE ↓ *Pde3b*, ↓ *Pde8a*, ↓ *Pde10a*	↑ insulin secretion	cAMP, Insulin secretion	Cells-β-Min6, HP62	[72]
Cocoa catchechins (25 µg/mL)	↑ *Hmox1*, ↑ *Nqo1*, ↑ *Nrf1*, ↑ GABPA,	↑ Mitochondrial electron complexes	Electron transport chain	Cells-INS-1 832/13	[76]
Tyrosol (25,50 µM/mL)	↓ GRP78, ↓ PERK, ↓ *eIFα*, ↓ *CHOP*↓ *XBP-1*, ↓ p-JNK	↓ Apoptosis, ↑ β-cells survival	JNK	Cells-NIT-1,	[77]
Jojoba seed extracts (150 µg/mL)	↑ *Nfr2*, ↓ *p22phox*, ↓ Casp-3, ↑ SOD & CAT	↓ ROS/OS	Mitochondrial	Cells-RINm5f	[78]
γ-Oryzanol (0.2 or 2.0 µg/mL)	↓ *Dnajb9*, ↓ *Xbp1s*, ↓ *Chop*, ↓ Casp3, ↓ CAD	↑ β-cell function, ↓ ER stress	ER Stress	Cells-MIN6	[79]
Epigallocatechin gallate (1–10 µM)	↑ *Pdx-1*, ↑ *FOXO1* ↑ pAkt	↑ Β-cell function, ↑ insulin secretion	pAkt/Pdx-1	Cells-RIN-m5F	[80]
Centratherum anthelminticum seeds (6.25–50 µg/mL)	↑ *Glut2*,	↑ β-cell function	Insulin secretion	Cells-β-TC6,	[81]
**In vivo model**					
γ-Oryzanol (320 µg/g BW)	↓ *Dnajb9*, ↓ *Xbp1s,* ↓ *Chop*, ↓ Casp3, ↓ CAD	↑ β-cell function, ↓ ER stress	ER Stress	Pancreaticisliets-C57BL/6J mice,	[79]

SIRT1—Sirtuin 1; Glut2—glucose transporter 2; GK—glucokinase; Pdx-1—pancreatic and duodenal homebox 1; Hnf-1α—hepatocyte nuclear factor 1 alpha; Tfam—mitochondrial transcription factor A; cAMP—Cyclic adenosine 3′,5′-monophosphate; PDE—Phosphodiesterase; Hmox1—hemeoxygenase 1; Nqo1—NAD(P)H quinone oxidoreductase 1; Nrf1/Nrf2—Nuclear respiratory factor 1 and 2; GABPA—GA binding protein transcription factor alpha subunit; GRP78—78-kDa glucose-regulated protein; PERK—Protein kinase-like endoplasmic reticulum kinase; eIFα—Inositol-requiring kinase alpha; CHOP—C/EBP-homologous protein; XBP-1—X box binding protein 1; p-JNK—Phosphorylated c-Jun N-kinase; p22phox—Neutrophil cytochrome b 22 kDa polypeptide; SOD—Super oxide dismutase; CAT—Catalase; Dnajb9—DnaJ homolog subfamily B member 9; Casp-3—Caspase 3; CAD—Caspase-activated DNase; FOXO1—Forkhead box O1; pAkt—Phosphorylated protein kinase A; ↑—increased gene expression; ↓—decreased gene expression.

**Table 3:** Polyphenols and gene modulations on insulin signalling pathways.

Polyphenols/Conc.	Gene Affected	Function	Pathways	Cells/Tissue Type	Ref.
**In vitro models**					
OSPT (500 µg/mL) and OSPL (100 µg/mL)	↑ *Glut4*, ↑ *Nrf1*, ↑ *Mef2a*, ↓ *Acc2*.	↓ Hyperinsulinemia, ↓ Lipid peroxidation	Insulin sensitivity	Cells-C2C12	[94]
Strigolactone GR24 and pinosylvin (60–100 µM)	↑ *SIRT1*, ↑ *Glut4* ↑ *FOXO1* ↑ IRS-1 ↑ Akt2,	↑ Insulin sensitivity, ↑ Glucose uptake	AKt2	Cells-L6 myoblasts	[100]
Hibiscus sabdariffa (Various dose)	↑ IRS-1, ↑ PI3K, ↓ DPP4, ↓ GLP-1R	↑ Insulin sensitivity, ↓ Starch breakdown	Insulin receptor activation (PI3K)	Cells-HK-2	[101]
C3G and PCA (10–100 µmol)	↑ PPARγ, ↑ *Glut4*, ↑ Adiponectin	↑ Glucose uptake	PPARγ	Cells-3T3-L1	[10]
Rice bran extracts (10 μg/mL and 50 μg)	↑ *PPARγ*, ↑ Adiponectin ↓ TNF-α	↑ Insulin sensitivity	PPARγ/adipogenesis	Cells-3T3-L1	[102]
Pigmented rice bran extracts (50 µg/mL)	↑ INSR, ↑ PI3K, ↑ *Glut4*, ↓ DDP-4	↑ Insulin sensitivity, ↓ Starch breakdown	Akt2/PI3K	Cells-3T3-L1	[103]
Aspalathin (1 µM)	↑ *Glut4,* ↑ UCP2, ↓ CPT1, ↑ *Bcl-1*	↑ Cell viability, ↑ Insulin sensitivity, ↑ Glucose uptake	pAMPK	Cells-H9c2	[104]
**In vivo models**					
Polyphenol-rich ethyl acetate fraction (200 mg/kg BW)	↑ Insr, ↑ IRS1, ↑ IRS2 ↑ Akt2, ↑ *Glut4*	↑ Insulin sensitivity	IRS1/AKT	Skeletal muscle-Sprague-Dawley rats	[105]
Folium Mori Extract (2 g/kg BW)	↑ IRS-1, ↑ PI3Kp85α, ↑ Glut-4	↑ Glucose uptake	IRS-1/PI3K/Glut-4 signalling	Skeletal muscle- Sprague-Dawley rats	[106]

Glut4—Glucose transporter 4; Nrf1—Nuclear respiratory factor 1; Mef2a—Myocyte enhance factor 2A; ACC2—Acetyl CoA carboxylase 2; SIRT1—Sirtuin 1; FOXO1—Forkhead box O1; IRS-1—Insulin receptor substrate 1; Akt2—Protein kinase B; PI3K (p85α phosphorylated)—Phosphatidylinositol 3-kinase; DPP4—Dipeptidyl-peptidase-4; GLP-1R—Glucagon-like peptide 1 receptor; PPARγ—peroxisome proliferator-activated receptor gamma; TNF-α—Tumor necrosis factor alpha; INSR—insulin substrate receptor; UCP2—uncoupling protein 2; CPT1—carnitine palmitoyltransferase-1; Bcl-1—B Cell Lymphoma 1; IRS1—insulin receptor substrate 1; ↑—increased gene expression; ↓—decreased gene expression.

**Table 4:** Polyphenols and gene modulations on gluconeogenesis pathways.

Polyphenols	Genes Affected	Function	Pathways	Cells/Tissue Type	Ref.
***In vitro* models**					
Cinnamon extract (1–25 µg/mL)	↓ PEPCK, ↓ *G6Pase*	↓ Hepatic glucose output	PEPCK	Cells-H4IIE	[126]
Fructus Corni (50 mg/mL)	↓ PEPCK	↓ Hepatic Glucose out put	Gluconeogenesis	Cells-H4IIE	[128]
EGCG (5–25 µM)	↓ PEPCK, ↓ G6Pase	↓ Hepatic glucose out put	PI3K	Cells-H4IIE	[57]
EGCG (≤1–10 µM)	↓ PEPCK, ↓ *G6Pase*	↓ Hepatic glucose output	AMPK/CaMKK	Cells-H4IIE	[130]
Germinated black rice (50 ppm)	↓ Pck1, ↓ Fbp1	↓ Hepatic glucose output	Gluconeogenesis	Cells-HepG2	[134]
**In vivo models**					
Germinated black rice (50–100 ppm)	↓ Pck1, ↓ Fbp1	↓ Hepatic glucose output	Gluconeogenesis	Liver-Sprague-Dawley rats	[134]
Hesperidin and Naringin (0.2 g/kg BW)	↓ *G6Pase*, ↓ PEPCK	↓ Hepatic glucose output	Gluconeogenesis	Liver-C57BL/KsJ-*db/db* mice	[136]
Resveratrol (0.02% *w*/*w*)	↓ PECK, ↓ G6P, ↑ GK, ↓ *SREBP-1c*	↑ Hepatic glucose uptake	PEPCK/AMPK	Liver-C57BL/KsJ-*db/db* mice	[137]
Fermented food paste (0.1–1.0 kg/BW)	↑ G6PD, ↑ *GCK*, ↑ PFK, ↑ 6PGD	↑ Glycogen synthesis, ↑ Hepatic insulin sensitivity, ↓ Hepatic glucose output	Glycolysis	Liver-Balb/c mice	[138]

PEPCK—Phosphoenolpyruvate carboxykinase; G6Pase—Glucose-6-phosphatase; Pck1—Phosphoenolpyruvate carboxykinase 1; Fbp1—Fructose-1,6-bisphosphatase 1; GK—glucokinase; SREBP-1c—sterol regulatory element-binding protein-1c; G6PD—glucose-6-phosphate dehydrogenase; GCK—glucokinase gene; PFK—Phosphofructokinase; 6PGD—6-Phosphogluconate dehydrogenase deficiency; AMPK—Adenine monophosphate activated protein kinase; CaMKK—Ca2+/calmodulin-dependent protein kinase kinase; ↑—increased gene expression; ↓—decreased gene expression.

## Abbreviations

6PGD	6-phosphogluconate dehydrogenase
*ACC2*	Acetyl CoA carboxylase 2
ACO	acyl-CoA oxidase
ADP	Adenosine diphosphate
Akt	Protein kinase B
AMPK	Adenine monophosphate activated protein kinase
ATP	Adenosine triphosphate
BIP	Binding immunoglobulin protein
C3G	Cyanidin 3-glucoside
CAD	Caspase-activated DNase
CAMFs	Methanolic fraction of *C. anthelminticum* seeds
CaMKK	Ca2+/calmodulin-dependent protein kinase kinase
cAMP	Cyclic adenosine 3′,5′-monophosphate
CAT	Catalase
Cdkn1a	Cyclin-dependent kinase inhibitor 1A
cDNA	Complementary deoxyribonucleic acid
CHOP	C/EBP-homologous protein
COX-2	cyclooxygenase-2
CPT-1	carnitine palmitoyltransferase-1
Dnajb9	DnaJ homolog subfamily B member 9
DPP-4	Dipeptidyl-peptidase-4
EGCG	Epigallocatechin gallate
eIF2α	Inositol-requiring kinase 2α
ENOS	Endothelial nitric oxide synthase
ER	Endoplasmic reticulum
ERdj4	ER resident DNAJ 4
FABP4	fatty acid binding protein 4
FADH	Flavin adenine dinucleotide
Fbp	Fructose-1,6-bisphosphatase
FBS	Fetal bovine serum
FFAs	Free fatty acids
FFP	Fermented food paste
FOXO1	Forkhead box O1
FRB	Fermented rice bran
G6Pase	Glucose-6-phosphatase
GABPA	GA binding protein transcription factor alpha subunit
Glut2	Glucose transporter 2
Glut4	Glucose transporter 4
Gpx	Glutathione Peroxidase
GRP78	78-kDa glucose-regulated protein
GSH	Glutathione
GSIS	Glucose stimulated insulin secretion
HDACs	histone deacetylases
Hmox1	hemeoxygenase 1
ICAM1	intracellular adhesion molecules-1
IGFR	Insulin-like growth factor receptor
IL-1β	Interleukin 1β
IL-6	Interleukin 6
iNOS	Inducible nitric oxide synthase
Ins1	Insulin 1
IR-β	Insulin receptor β
IRS2	Insulin receptor substrates 2
JAK/STAT	Janus kinase/signal transducer and activator of transcription
JNK	c-Jun N-kinase
LPH	Lactase phloridzin hydrolase
LXRα	Liver X receptor alpha
Mafa	MAF BZIP Transcription Factor A
MAPK	Mitogen-activated protein kinase
MEF2a	Myocyte enhance factor 2A
NADH	Nicotinamide adenine dinucleotide
NADPH	Nicotinamide adenine dinucleotide phosphate
NF-kB	Nuclear factor kappa light chain enhancer of activated B cells
Ngn3	Neorog-3
NO	Nitric oxide
Nqo1	NAD(P)H quinone oxidoreductase 1
Nrf2	Nuclear factor erythroid 2-related factor 2
p22phox	Neutrophil cytochrome b 22 kDa polypeptide
P3G	Peonidin 3-glucoside
pAkt	Phosphorylated protein kinase A
PARP	Poly ADP-ribose polymerase
Pck	Phosphoenolpyruvate carboxykinase
*Pck1*	Phosphoenolpyruvate carboxykinase 1
Pde	Phosphodiesterase
Pdx-1	Pancreatic andduodenal homeobox 1
PEPCK	Phosphoenolpyruvate carboxykinase
PERK	Protein kinase-like endoplasmic reticulum kinase
PFK	Phosphofructokinase
PI3K	Phosphatidylinositol 3-kinase
pIRS-1	Phosphorylated insulin receptor substrate 1
PKA	Protein kinase A
PPAR	peroxisome proliferator-activated receptor
RNA	Ribonucleic acid
RNS	Reactive nitrogen species
ROS	Reactive oxygen species
RTK	Receptor tyrosine kinases
Sirt1	Sirtuin 1
Socs3	Suppressor of cytokine signalling 3
SOD2	Superoxide dismutase 2
SREBP-1c	sterol regulatory element-binding protein-1c
T2DM	Type 2 diabetes mellitus
TCA	Tricarboxylic acid
Tfam	Mitochondrial transcription factor A
TGFβ1	Transforming growth factor-β1
TNF-α	*Tumour necrosis factor alpha*
Tnfsf	Tumour necrosis factor receptor superfamily
TXNIP	Thioredoxin interacting protein
UPR	Unfolded protein response
Xbp1s	X box binding protein 1
